# A Pseudovirus Nanoparticle Displaying the Vaccinia Virus L1 Protein Elicited High Neutralizing Antibody Titers and Provided Complete Protection to Mice against Mortality Caused by a Vaccinia Virus Challenge

**DOI:** 10.3390/vaccines12080846

**Published:** 2024-07-26

**Authors:** Pengwei Huang, Ming Xia, Frank S. Vago, Wen Jiang, Ming Tan

**Affiliations:** 1 Division of Infectious Diseases, Cincinnati Children’s Hospital Medical Center, 3333 Burnet Avenue, Cincinnati, OH 45229, USA; pengwei.huang@cchmc.org (P.H.); ming.xia@cchmc.org (M.X.); 2 Department of Biological Sciences, Purdue University, West Lafayette, IN 47907, USA; fvago@purdue.edu (F.S.V.); jiang12@purdue.edu (W.J.); 3 Department of Pediatrics, University of Cincinnati College of Medicine, Cincinnati, OH 45229, USA

**Keywords:** mpox, vaccinia, smallpox, vaccine, norovirus S particle, pseudovirus nanoparticle, nanoparticle vaccine

## Abstract

The recent worldwide incidence of mpox infection and concerns about future emerging variants of mpox viruses highlight the need for the development of a new generation of mpox vaccines. To achieve this goal, we utilized our norovirus S nanoparticle vaccine platform to produce and evaluate two pseudovirus nanoparticles (PVNPs), S-L1 and S-J1. These PVNPs displayed the L1 neutralizing antigen target of the vaccinia virus and a yet-untested J1 antigen of the mpox virus, respectively, with the aim of creating an effective nanoparticle-based mpox vaccine. Each self-assembled PVNP consists of an inner shell resembling the interior layer of the norovirus capsid and multiple L1 or J1 antigens on the surface. The PVNPs improved the antibody responses toward the displayed L1 or J1 antigens in mice, resulting in significantly greater L1/J1-specific IgG and IgA titers than those elicited by the corresponding free L1 or J1 antigens. After immunization with the S-L1 PVNPs, the mouse sera exhibited high neutralizing antibody titers against the vaccinia virus, and the S-L1 PVNPs provided mice with 100% protection against mortality caused by vaccinia virus challenge. In contrast, the S-J1 PVNPs induced low neutralizing antibody titers and conferred mice weak protective immunity. These data confirm that the L1 protein is an excellent vaccine target and that the readily available S-L1 PVNPs are a promising mpox vaccine candidate worthy of further development.

## 1. Introduction

Mpox (formerly known as monkeypox) is a zoonotic infectious viral disease caused by the mpox virus that causes symptoms similar to those observed in smallpox patients, although these infections are less clinically severe. Typical signs of mpox include skin rash and mucosal lesions accompanied by fever, headache, and, in severe cases, even death. Both mpox and its closely related counterpart, smallpox, are listed as diseases with epidemic or pandemic potential by the World Health Organization (WHO). Smallpox and mpox are also considered potential bioterrorism agents [1].

Historically, mpox was found to be endemic in central and western Africa, where several species of mammals are believed to be natural reservoirs of the virus. However, a recent outbreak starting in May 2022 documented approximately 91,788 confirmed cases in 116 countries, resulting in 167 deaths. This mpox outbreak was caused by clade IIb mpox viruses [2]. Notably, 85,525 cases occurred in locations that had not historically reported mpox disease, including 30,347 cases in the United States and 26,227 in Europe [3]. Most recently, a surge of mpox in the Democratic Republic of the Congo, resulted from clade I mpox viruses, has caused nearly 13,000 suspected cases and 581 suspected deaths [2], raising serious concerns about another global mpox outbreak that may result in more deaths than the one starting in 2022. Therefore, mpox has emerged as a global public health threat.

Like smallpox virus and vaccinia virus, the mpox virus belongs to the Orthopoxvirus genus in the Poxviridae family, a group of double-stranded DNA viruses. Like in other poxviruses, an mpox virion is barrel-shaped, with dimensions of approximately 360 × 270 × 250 nm. Each virion contains a linear, double-stranded DNA genome encoding approximately 250 genes [4]. The virions exist in two antigenically distinct forms: intracellular mature virions (IMVs) and extracellular enveloped virions (EEVs). For infection, the virus particle attaches to the host cell through recognition of cell surface glycosaminoglycans [5] and then enters the host cells through various viral–host protein interactions [4]. The mpox viruses reproduce in the cytoplasm using host ribosomes to generate the structural components of virions. Progeny poxviruses are enveloped by membranes before exiting infected cells via plasma membrane fusion or by cell rupture.

The peripheral proteins of the vaccinia virus have been extensively studied, revealing approximately 25 membrane proteins [6]. Among them, 17 are suggested to play a functional role in viral penetration and/or virus-host cell membrane fusion. These proteins include A27, A17, H3, D8, L1, A28, H2, A21, L5, G3, G9, A16, J5, F9, I2, A26, and O3 [7]. The entry–fusion complex comprises eight proteins: A16 [8], A21 [9], A28 [10], G3 [11], G9 [11], H2 [12], L5 [13], and O3 [14]. The membrane proteins A27 and H3 facilitate intracellular IMV virion attachment to host cells via interactions with cell surface glycosaminoglycans [15,16,17]. Immunization with the vaccinia vaccine in humans elicits antibodies against four major IMV surface proteins: A27, L1, D8, and H3 [18,19]. To date, six membrane antigens of vaccinia viruses, namely A27, A33, L1, D8, B5, and H3, are known to be important targets for protective immune responses [20]. The crystal structures of the L1, A33, and D8 proteins have been elucidated [21,22,23]. In particular, the structures of L1 and D8 in complex with corresponding neutralizing antibodies were solved, with L1 binding to the monoclonal antibody (mAb) 7D11 [24] and D8 binding to the mAb LA5 [21].

Due to their close genetic and antigenic relationships with mpox and smallpox viruses, less virulent vaccinia viruses are employed as vaccines against these two pathogens. For instance, Jynneos™ (Bavarian Nordic, Kvistgård, Denmark) contains a live, modified, nonreplicating vaccinia virus Ankara strain that provides ~85% effectiveness against mpox [25,26,27,28,29]. Due to the nature of these live vaccinia viruses, one of the currently licensed mpox/smallpox vaccines, ACAM2000, which comprises live replicating vaccinia viruses, is associated with a relatively high rate of adverse events in healthy persons. These include the risk of peri- and endocarditis, severe debilitation, and even death [30], posing a particular high risk to immunocompromised individuals and pregnant women [1,31]. The Jynneos™ vaccine, which consists of nonreplicating vaccinia virus, removes the observed pathologies associated with the ACAM2000 vaccine and is therefore much safer. However, Jynneos™ still has common side effects like injection site reactions and systemic symptoms such as headache and muscle pain. Additionally, future emerging variants of mpox virus may not be fully covered by existing vaccines. New vaccines may also be developed to target specific strains or clades of the mpox virus, providing more tailored protection. Finally, more effective vaccines with simpler administration processes will improve accessibility and distribution, particularly in resource-limited developing countries. Therefore, there is a high demand for a new generation of mpox vaccines with improved effectiveness and safety. To address this public health need, several candidates of DNA vaccines, mRNA vaccines, as well as recombinant protein vaccines containing multicomponents of a subset of IMV and EEV antigens, were generated and evaluated, demonstrating protection against orthopoxvirus challenges in mice and nonhuman primates [32,33,34,35,36]. Several IMV surface proteins have been shown to be neutralizing antigens [7,37], and the IMV antigen L1 as a single target has also been shown to protect mice from vaccinia virus challenge [38]. Consequently, L1 is frequently included in subunit vaccine candidates [32,33,34,35].

Recently, we developed a norovirus inner shell (S)-based nanoparticle as a vaccine platform to create pseudovirus nanoparticles (PVNPs) displaying various antigens in polyvalent formats, aiming to enhance immune responses [39,40,41,42]. When the norovirus S domain (22.97 kDa) is produced using a protein-expressing system, the S protein self-assembles into 22 nm S nanoparticles (SNPs) with T = 1 icosahedral symmetry, consisting of 60 S proteins [40]. Each SNP has 60 exposed, flexible S domain termini on the surface, making the SNP an excellent platform for displaying antigens of other pathogens. In this study, we generated two self-assembled PVNPs, each of which displayed the L1 antigen of vaccinia virus or the J1 antigen of mpox virus, and evaluated them as vaccine candidates. L1 is a myristoylated peripheral protein of vaccinia virus that plays a key role in facilitating viral–host cell membrane fusion and viral entry [43], two critical initial steps in mpox/vaccinia virus infection. Thus, L1 is a major neutralizing antigen and an ideal target for mpox vaccine development [24,38]. On the other hand, J1 is a membrane protein essential for virion morphogenesis, growth, and plaque formation [44]. Unlike the L1 protein, which has been proven to be an excellent vaccine target among several DNA/subunit vaccine candidates, J1 has not yet been studied as a vaccine target. Both the L1 and J1 proteins are highly conserved among orthopoxviruses, with amino acid sequences nearly identical between mpox and vaccinia viruses.

Both types of PVNPs self-assemble after recombinant S-L1 and S-J1 fusion proteins are generated through the use of the mammalian CHO (Chinese hamster ovary) cell expression system. While both types of PVNPs were immunogenic in mice, eliciting high antibody titers toward the displayed L1 or J1 antigens, only the sera from mice immunized with the S-L1 PVNPs demonstrated a high neutralizing antibody titer against vaccinia virus replication. Additionally, the S-L1 PVNPs provided mice with 100% protection against mortality caused by vaccinia virus challenge. Thus, the S-L1 PVNPs stand out as a promising vaccine candidate against poxviruses.

## 2. Materials and Methods

**Plasmids for recombinant protein expression.** A DNA fragment was designed to encode the S-L1 fusion protein, which comprises the following sequence components: (1) an N-terminal secretory signal peptide (MKWVTFISLLFLFSSAYS) [39]; (2) a GII.4 norovirus S domain with an R69A mutation and a hinge, as described previously [40,41]; (3) a four-glycine linker (GGGG); (4) a polypeptide containing the first 184 amino acids of the L1 protein of vaccinia virus (Western Reserve strain, PDB ID: 4U6H); and (5) a C-terminal Hisx6 tag for purification purposes. Another DNA fragment encoding the S-J1 fusion protein was designed similarly, replacing the L1-encoding sequence with the J1 protein-encoding sequence (152 residues) of an mpox virus (GenBank AC#: YP_010377082.1). Both DNA fragments were synthesized by GenScript (Piscataway, NJ, USA) and inserted into the pcDNA^TM^3.4 vector (Thermo Fisher Scientific, Waltham, MA, USA) for protein expression in the CHO cell system, as described elsewhere [39]. Additionally, the DNA sequences encoding the L1 and J1 proteins were inserted into the pET24b vector, respectively, for protein expression using the *Escherichia coli* system.

**Recombinant protein expression and purification.** Recombinant S-L1 and S-J1 fusion proteins were expressed using the ExpiCHO Expression System (Gibco/Thermo Fisher Scientific, Waltham, MA, USA) through transient transfection as described in our previous study [39]. Briefly, ExpiCHO-S cells, prepared according to the manufacturer’s instructions at 6 × 10^6^ cells/mL, were transfected with the respective plasmids (see above) and incubated at 37 °C on an orbital shaker. At 24 h post transfection, the ExpiFectamine CHO Enhancer and ExpiCHO Feed were added. The cell cultures were harvested 10 days post transfection by centrifugation at 3450× *g* (5000 rpm) for 20 min at 4 °C using an Avanti J-26XP centrifuge (Beckman Coulter, Brea, CA, USA) with a JA14 rotor, after which the cells/cell debris were separated from the culture medium (supernatant). The culture supernatant was mixed with Hisx6 tag binding cobalt resin (Thermo Fisher Scientific) for three hours, after which the mixture was loaded into an empty column to collect the resin. After washing three to four times with 10 resin volumes of washing buffer (20 mM of sodium phosphate, 0.5 M of NaCl, pH 7.4), the bound target proteins were eluted using elution buffer (20 mM of sodium phosphate, 0.5 M of NaCl, 500 mM of imidazole, pH 7.4). Additionally, the S-L1 and S-J1 fusion proteins were produced using the CHO cell expression system through the protein expression service provided by GenScript.

In addition, C-terminally His-tagged L1 and J1 proteins were produced by the *E. coli* expression system using the BL21/DE3 Arctic strain and purified through a denaturing protocol following the instructions outlined in the HisPurTM Cobalt Resin Manual (Thermo Fisher Scientific), as described previously [39]. Briefly, after overnight induction with 0.4 mM of IPTG (isopropyl ß-D-1-thiogalactopyranoside), the bacteria were harvested and mixed with denaturing buffer (6 M of guanidine hydrochloride, 200 mM of tributylphosphine, 0.5 M of iodoacetamide, 100 mM of ammonium bicarbonate, 0.2 of mg/mL Proteomics Grade Trypsin; pH 8.5). Following sonication, the denatured target proteins were isolated using cobalt resin. The purified proteins were then serially dialyzed overnight against phosphate buffer (50 mM of sodium phosphate, 300 mM of NaCl, pH 7.4) containing 6 M, 4 M, 2 M, or 1 M of urea to remove the guanidine hydrochloride and reduce the concentration of urea. Finally, the proteins in phosphate buffer with 1 M of urea were dialyzed against redox buffer (100 mM of Tris, 400 mM of L-arginine, 2 mM of EDTA, 5 mM of reduced glutathione, and 1 mM of oxidized glutathione, pH 8.0) overnight to remove the urea for protein refolding.

**SDS-PAGE, protein quantitation, and Western blotting.** The quality of the purified recombinant proteins was evaluated through SDS-PAGE (sodium dodecyl sulfate-polyacrylamide gel electrophoresis) using 10% to 12% separating gels. Proteins were quantitated by SDS-PAGE using serially diluted bovine serum albumin (BSA; Bio-Rad, Hercules, CA, USA) at known concentrations as standards on the same gels [45]. The determined protein concentrations were cross-referenced with the absorbance at 280 nm using a NanoDrop Microvolume Spectrophotometer (Thermo Fisher Scientific). Western blotting was performed as described previously [39] using guinea pig hyperimmune sera against norovirus VLPs [46] containing the S domain. The secondary antibody used was a fluorescence-labeled donkey antibody against guinea pig IgG (IRdye 680RD, LI-COR Biosciences, Lincoln, NE, USA). Specific signals for the S-L1 and S-J1 proteins were documented using an Odyssey CLx imager (LI-COR Biosciences).

**Transmission electron microscopy (TEM).** The formation of PVNPs by the S-L1 or S-J1 fusion protein was examined using TEM, as described previously [40]. The purified S-L1 and S-J1 fusion proteins were applied to grids (TED PELLA, Inc., Redding, CA, USA) and stained with 1% ammonium molybdate. The air-dried grids were observed using an EM10 C2 electron microscope (Zeiss, Oberkochen, Germany) at 80 kV with magnifications ranging from 15,000× to 40,000×.

**Dynamic light scattering (DLS).** DLS was used to measure the size distribution of the S-L1 and S-J1 PVNPs following previously described methods [47]. In brief, the purified S-L1 and S-J1 proteins (200 µL each) were loaded into separate wells of a 96-well microplate with a clear bottom (Greiner Bio-One, Frickenhausen, Germany) and analyzed using a DynaPro Plate Reader III DLS instrument (Wyatt Technology, Santa Barbara, CA, USA). The recorded data were then analyzed using the software DYNAMICS version 8 (Wyatt Technology).

**Structural modeling of the S-L1 PVNPs.** UCSF ChimeraX software (version 1.4) [48] was used to construct 3-dimensional (3D) structural models of S-L1 PVNPs utilizing the cryoEM (cryogenic electron microscopy) density map of the S_60_-VP8* nanoparticles [40] as a template. The crystal structures of the inner shells of the 60-valent VLPs of feline calicivirus (FCV) (PDB code: 4PB6) [49] or GII.4 NoV [39], and the L1 protein of vaccinia virus (PDB code: 1ypy) [22] were incorporated into the corresponding regions of the template. UCSF ChimeraX software was also used for structural analysis of the S-L1 model and for creation of the final images.

**Mouse immunization for L1/J1-specific antibody response.** Forty pathogen-free BALB/c mice aged ~6 weeks were randomly divided into five groups of 8 mice each (n = 8 mice/group). The mice in each group were immunized with one of the following immunogens at 10 µg/mouse three times at two-week intervals: (1) S-L1 PVNP (S-L1); (2) S-J1 PVNP (S-J1); (3) free L1 protein (L1); (4) free J1 protein (J1); and 5) S_60_ nanoparticle (S) as a negative control. All immunogens were adjuvanted with aluminum salt (Imject Alum, Thermo Fisher Scientific) at 25 μL/dose (20 μg/mouse/dose). Immunogens were administered intramuscularly (IM) in the thigh muscle three times in an 80 μL volume. Small amounts of blood samples were taken two weeks after the second immunization through the tail vein, while blood was collected two weeks after the third immunization via heart puncture. Sera were prepared from blood samples through an established protocol [50]. All mice were purchased from the Jackson Laboratory (Bar Harbor, ME, USA) and were maintained at the Division of Veterinary Services of Cincinnati Children’s Hospital Medical Center (CCHMC) during the experiments.

**Determination of serum antibody titers specific to L1 or J1 antigen.** L1- and J1-specific IgG and IgA titers were measured using an enzyme immunoassay (EIA). Briefly, 96-well microtiter plates were coated with bacterially expressed and purified L1 or J1 protein at a concentration of 5 µg/mL as capture antigens. After blocking with 5% nonfat milk, mouse sera were serially diluted and incubated with the coated antigens. The detection of bound IgG was performed using a goat-anti-mouse IgG-HRP (horseradish peroxidase) conjugate (1:5000, MP Biomedicals, Solon, OH, USA) for L1/J1-specific IgG. For L1/J1-specific IgA, a goat-anti-mouse IgA-HRP conjugate (1:2000, Invitrogen, Waltham, MA, USA) was used. The L1/J1-specific IgG/IgA titers were defined as the maximum dilutions of sera that produced positive signals (OD450 ≥ 0.2).

**Serum-neutralizing antibody titers against vaccinia virus replication.** These titers were determined by the plaque reduction neutralization test (PRNT) as described elsewhere [38]. Briefly, Vero E6 cells were grown in 6-well culture plates until confluence. Vaccinia virus Western Reserve (WR) strain (kindly provided by Dr. Koichi Araki at Cincinnati Children’s Hospital Medical Center) at ~150 PFU in Dulbecco’s modified Eagle’s medium (DMEM) was treated with 2-fold serially diluted mouse serum for 30 min at 37 °C in a total volume of 0.5 mL. The virus/serum mixture was subsequently added to the cells. Wells with cells and vaccinia virus without serum treatment served as positive controls, while wells with cells and medium only without virus/serum served as negative controls. After the plates were shaken gently for 1.5 h at 37 °C, the virus/serum mixture was removed, and the cells were overlaid with 2 mL of DMEM containing 2% FBS. After 4 days of incubation at 37 °C, the cells were fixed with 3.7% formaldehyde and stained with a 0.5% crystal violet solution for plaque counting. Serum-neutralizing titers were defined as the maximum dilutions of the sera that showed at least 50% plaque reduction compared with the positive control wells.

**Mouse vaccination and vaccinia virus challenge.** Twenty-four female BALB/c mice at 6 weeks of age were randomly divided into three groups (n = 8 mice/group). The mice in each group were immunized intramuscularly with one of the following immunogens at 10 µg/mouse three times at two-week intervals: (1) S-L1 PVNP (S-L1), (2) S-J1 PVNP (S-J1), or (3) S_60_ nanoparticle (S). Two weeks after the third immunization, mouse blood samples were collected via the tail vein for L1/J1-specific antibody titer determination. Sixteen days after the third immunization, the mice were intranasally challenged with 4 times the median lethal dose (equivalent to 1.6 × 10^5^ PFU) of vaccinia virus (WR strain). The weight changes, disease progression, and survival rates of the challenged mice were monitored daily for 14 days. As required by IACUC guidelines, the challenged mice were euthanized when they lost more than 25% of their original body weight. The experiment was terminated after 14 days.

**Ethics statement.** All animal studies were conducted in compliance with the recommendations in the Guide for the Care and Use of Laboratory Animals (23a) of the National Institute of Health (NIH). The protocols used were approved by the Institutional Animal Care and Use Committee (IACUC) of the Cincinnati Children’s Hospital Research Foundation (animal welfare assurance No. A3108-01).

**Statistical analysis.** Statistical differences between two data groups were calculated by GraphPad Prism 9.0 (GraphPad Software, Inc., San Diego, CA, USA) via an unpaired *t* test. Differences in survival curves were analyzed employing the log-rank (Mantel–Cox) test (chi-square test). The statistical significance of the differences was determined as follows: nonsignificant (marked as “ns”) for *p* values > 0.05, significant (marked as “*”) for *p* values < 0.05, highly significant (marked as “**”) for *p* values < 0.01, and extremely significant (marked as “***”) for *p* values < 0.001, or (marked as “****”) for *p* values < 0.0001.

## 3. Results

**Figure 1:**
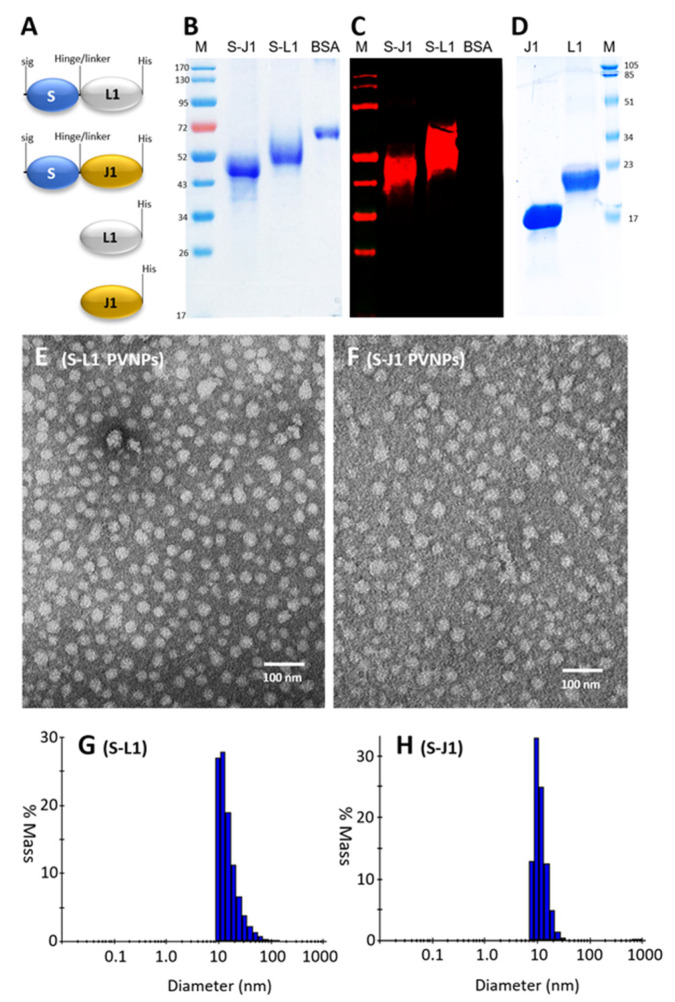
Generation and characterization of the S-L1 and S-J1 fusion proteins and their self-assembled PVNPs. (**A**) Schematic constructs of the S-L1 and S-J1 fusion proteins, as well as the free L1 and J1 proteins. Sig: secretory signal peptide; S: modified norovirus S domain; hinge: the hinge region of norovirus VP1; linker: a row of four glycines; L1/J1: the L1/J1 protein; His: the Hisx6 tag. (**B**) An SDS-PAGE gel showing the quality of the resin-purified S-J1 and S-L1 fusion proteins, each revealing major bands at ~45 kDa and ~52 kDa. Bovine serum albumin (BSA, ~68 kDa) served as a control. (**C**) A Western blot analysis of the purified S-J1 and S-L1 fusion proteins (each lane contained 0.25 µg of protein) was performed using hyperimmune serum against norovirus VLPs, which contain the S domain as a primary detection antibody. BSA was included as a negative control. (**D**) An SDS-PAGE gel showing the quality of the resin-purified J1 and L1 proteins, each revealing a major band at the expected ~17 kDa and ~20 kDa. In (**B**,**C**), the M lanes are protein standards with the indicated molecular weights in kDa. (**E**,**F**) TEM micrographs of the purified S-L1 (**E**) and S-J1 (**F**) proteins indicating PVNP formation. (**G**,**H**) The size distributions of the S-L1 (**G**) and S-J1 PVNPs were determined by dynamic light scattering (DLS).

**Generation and characterization of the S-L1 and S-J1 proteins.** C-terminally Hisx6-tagged, soluble S-L1 and S-J1 fusion proteins (Figure 1A) were produced using the mammalian CHO cell expression system and purified with Cobalt resin at a yield of ~20 mg/100 mL of CHO cell culture for the S-L1 protein and ~10 mg/100 mL of CHO cell culture for the S-J1 protein. SDS-PAGE analysis of the two purified proteins revealed a single major band each at a molecular weight (MW) of ~52 kDa (for S-L1) and ~45 kDa (for S-J1) (Figure 1B), which were larger than their calculated MWs of ~44.4 (for S-L1) and 41.7 (for S-L1) kDa, respectively. To prove the identities of the two proteins, a Western blot analysis was performed using a previously prepared antibody against norovirus VLPs that contain the S domain protein. The specific signals to the purified S-L1 and S-J1 proteins (Figure 1C) verified their identities. The observed mobility shifts of the two proteins in the SDS-PAGE analysis may represent their posttranslational modifications, including the known myristoylation of the L1 protein [51]. The smeared signals around the two major bands (Figure 1B,C) may represent protein molecules with more or fewer modifications than the majority of the proteins.

We also prepared the free L1 and J1 proteins without the S domain using the bacterial expression system at yields of 15 to 20 mg/L of bacterial culture. SDS-PAGE showed that the MWs were as expected at 19.5 (L1) and 17.3 (J1) kDa, respectively (Figure 1D). Since the *E. coli* system lacks posttranslational modifications, the less smear bands corresponding to the L1/J1 proteins on the SDS-PAGE gel further support that the mammalian cell-expressed S-L1 and S-J1 proteins retained their posttranslational modifications.

**Self-assembly of the S-L1 and S-J1 PVNPs.** Inspection of the purified S-L1 and S-J1 protein samples via TEM after negative staining revealed numerous nanoparticles, the majority of which were approximately 26 nm in diameter. This indicated that the S-L1 and S-J1 proteins self-assembled into PVNPs (Figure 1E,F), apparently due to the tendency of the norovirus S domain to form shell-like nanoparticles [40]. It was observed that some of the PVNPs were larger or smaller than 26 nm. Norovirus VLPs are known to be organized in T = 1, T = 3, and/or T = 4 icosahedral symmetry with different VLP sizes that are composed of 60, 180, and 240 VP1 proteins, respectively [52,53]. Thus, the larger PVNPs may represent those with T = 3 and T = 4 icosahedral symmetries, comprising 180 and 240 fusion proteins respectively, while the symmetries of those smaller ones remain to be determined. The size distributions of the PVNPs were also analyzed by DLS, revealing that the particle sizes of the S-L1 PVNPs ranged from 10 to 40 nm, while those of the S-J1 PVNPs ranged from 7 to 40 nm (Figure 1G,H), consistent with the particle sizes observed by TEM.

**Figure 2:**
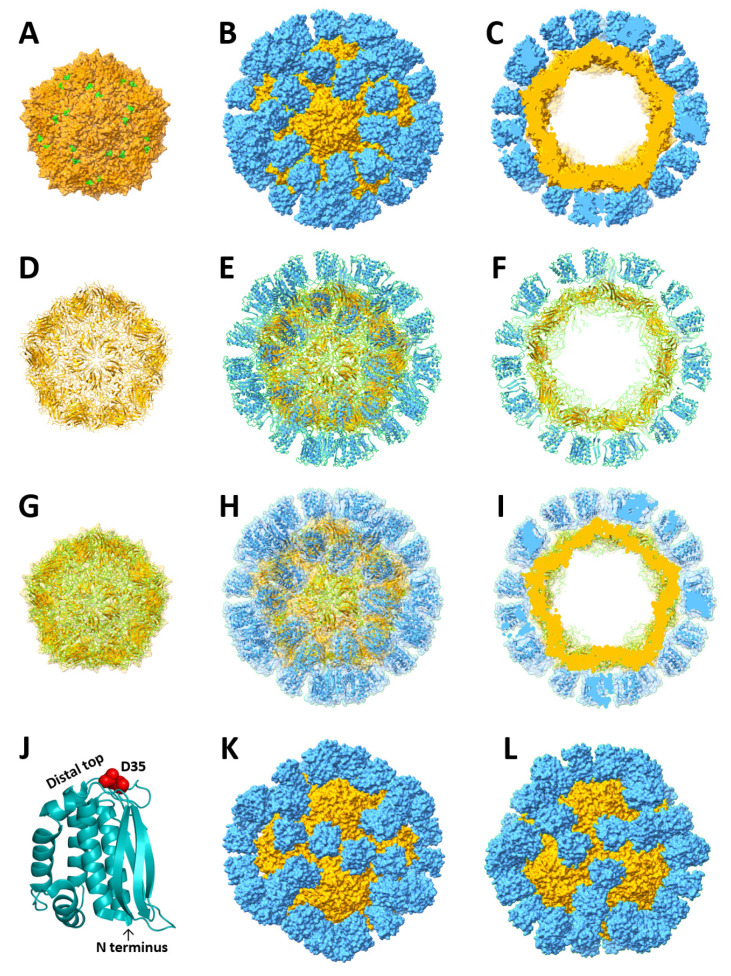
Three-dimensional (3D) structural modeling of the S_60_-L1 PVNP. With the help of UCSF ChimeraX software, a 3D model was generated using the previously elucidated cryoEM map of S_60_-VP8* PVNP [40], in which the N-termini of 60 L1 crystal structures (PBD code: 1ypy) [22] were docked to the C-termini of the S domains of the S_60_ nanoparticle to replace the VP8*s. (**A**) Surface representations of the S_60_ nanoparticle at the fivefold axis, showing 60 exposed S domain termini in green. (**B**,**C**) Surface representations of the S_60_-L1 PVNPs in the frontal (**B**) and section (**C**) views, respectively. (**D**–**F**) Cartoon representations of the above three S-L1 PVNP structures, including the S_60_ nanoparticle (**D**) and the front (**E**) and section (**F**) views of the PVNP. (**G**–**I**) Transparent surface representations showing the cartoon representations of the S-L1 PVNP, including its S_60_ nanoparticle (**G**), as well as the front (**H**) and section (**H**) views of the PVNP. (**J**) The crystal structure of the L1 antigen in cartoon representation showing its N-terminus at the base and residue D35 in the red sphere representation representing the major neutralizing epitope on the distal top of the antigen. (**K**,**L**) Surface representations of S_60_-L1 PVNPs viewed at twofold (**K**) and threefold (**L**) axes. In all the images, the S_60_ nanoparticles are shown in orange, while the L1 proteins are shown in cyan. All the images except (**J**–**L**) are viewed at the fivefold axes.

**3D structural modeling of the S_60_-L1 PVNP.** The major population of the S-L1 and S-J1 PVNPs, ~26 nm in diameter, resembled that of the S_60_-VP8* PVNPs, whose structure with T-1 icosahedral symmetry has been elucidated by cryoEM [40]. This strongly suggested that the majority of the S-L1 and S-J1 PVNPs shared the same T = 1 icosahedral symmetry and consisted of 60 S-L1/S-J1 proteins. Since the crystal structure of the L1 protein has been solved [22], we utilized the cryoEM structure of S_60_-VP8* PVNP [40] as a template to generate a 3D structural model of the S_60_-L1 PVNP for a deeper understanding of its structural features. Specifically, the S_60_-L1 PVNP model was created (Figure 2) using UCSF ChimeraX software by docking the N-terminal ends of the 60 L1 structures (PDB code: 1ypy) to the 60 exposed C-terminal ends of the S domains of the S_60_ nanoparticle (Figure 2A) based on the cryoEM density map of the S_60_-VP8* PVNP.

Based on this model, images representing the full and sectional views of the S_60_-L1 PVNP in the surface representations (Figure 2A–C), cartoon representations (Figure 2D–F), and transparent surface models showing the cartoon representations (Figure 2G–I), were generated. These image analyses indicated that 60 L1 proteins stand upright on the surface of the S_60_ nanoparticle, forming the S_60_-L1 PVNP. The previously identified conformational neutralizing epitope centering at ASP35 (D35) [38] is located at the distal end of each L1 protein (Figure 2J). This model also indicates that the PVNPs appear differently at various viewing angles. While it is round-shaped at the fivefold axis (Figure 2B,C,E,F,H,I), it is more angular-shaped with a round corner (Figure 2K) at the twofold axis and a pentagon with the top corner snipped (Figure 2L) at the threefold axis. This difference may explain the difference in the morphologies of the PVNPs observed in the TEM micrographs (Figure 1E,F). A 3D structural model of the S-J1 PVNPs was not generated due to the lack of the J1 structure.

**Figure 3:**
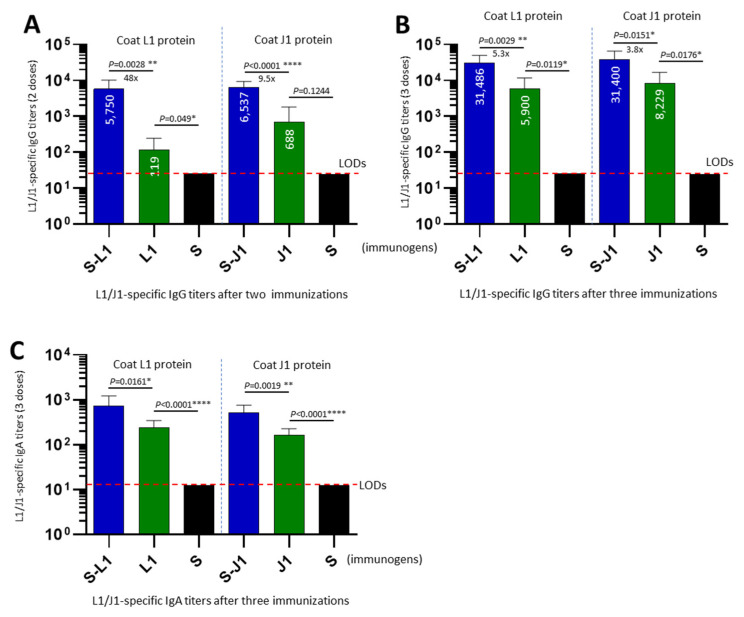
L1/J1-specific serum antibody responses elicited by the S-L1/S-J1 PVNPs in mice. (**A**,**B**) L1- and J1-specific serum IgG titers elicited by the S-L1 and S-J1 PVNPs, as well as by free L1 and J1 proteins after two (**A**) and three (**B**) immunizations. (**C**) L1- and J1-specific serum IgA titers elicited by the S-L1 and S-J1 PVNPs, as well as by free L1 and J1 proteins after three immunizations. The S_60_ nanoparticle (S) was used as a negative control. Y-axes indicate IgG (**A**,**B**) or IgA (**C**) titers, while X-axes indicate various immunogens. Purified L1 and J1 proteins were used as capture antigens for determination of the L1- (**left panels**) and J1-specific (**right panels**) IgG (**A**,**B**) and IgA (**C**) titers, respectively. Significant differences between the data sets are shown as “*” for significance with *p* values < 0.05, “**” for high significance with *p* values < 0.01, or “****” for extreme significance with *p* values < 0.0001. The dashed lines indicate the limits of detection (LODs).

**Enhanced immune responses to the PVNP-displayed L1 or J1 antigens.** To demonstrate the enhanced immune responses against the presented L1 and J1 antigens by polyvalent PVNPs, BALB/c mice were immunized with the S-L1 and S-J1 PVNPs, with free L1 and J1 proteins used as controls for comparison. EIA assays using the bacterially expressed and purified L1 or J1 protein as capture antigens revealed that after two or three immunizations, the S-L1 and S-J1 PVNPs induced significantly greater L1- or J1-specific serum IgG titers in mice compared to those elicited by the free L1 or J1 proteins, respectively (*P*s < 0.05; Figure 3A,B). Specifically, after three immunizations, the L1-specific IgG titer elicited by the S-L1 PVNPs reached 31,468, and the J1-specific IgG titer induced by the S-J1 PVNPs was 31,400, representing 5.3-fold and 3.8-fold increases, respectively, compared to those induced by the free L1 and J1 proteins, respectively. Serum IgA responses were also determined by EIAs, revealing a similar enhancement in L1- or J1-specific IgA responses by the S-L1 or S-J1 PVNPs compared to those elicited by the free L1 or J1 proteins. As a negative control, S_60_ nanoparticles did not elicit detectable L1- or J1-specific IgG or IgA titers. These data demonstrated that the S-L1 and S-J1 PVNPs substantially improved the antibody responses toward the displayed L1 and J1 antigens, respectively.

**Figure 4:**
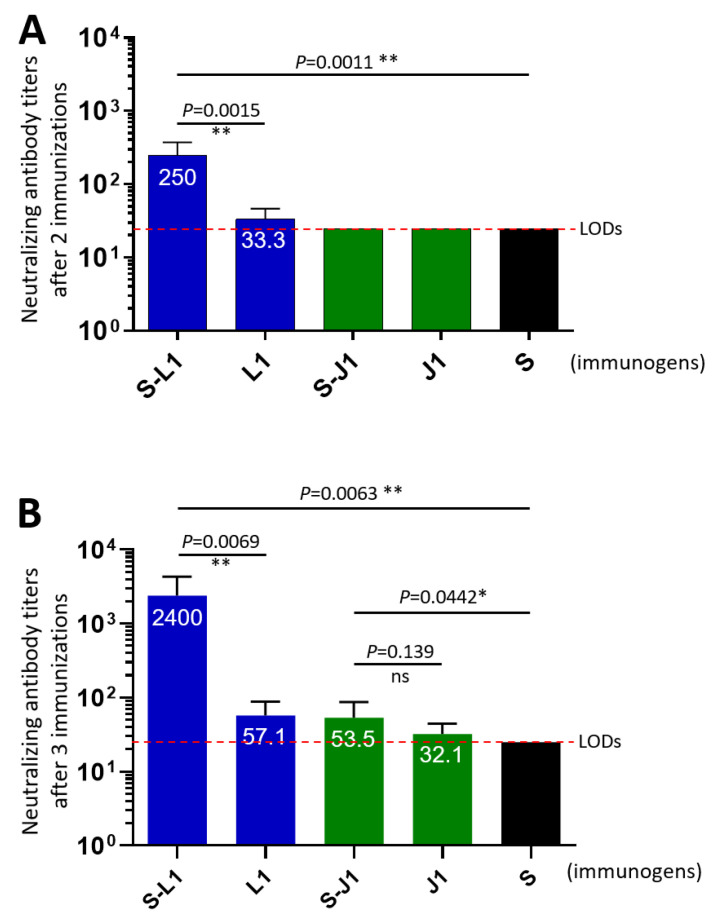
Neutralizing antibody titers of the mouse sera after immunization with the S-L1 PVNPs, the S-J1 PVNPs, the free L1 protein, or the free J1 protein for two (**A**) or three (**B**) times against vaccinia virus (Western Reserve strain) replication in cell culture. Y-axes show 50% neutralizing antibody titers of mouse sera, whereas X-axes indicate the mouse serum samples after immunization with various immunogens. The sera after immunization with the S_60_ nanoparticles (S) served as negative controls. Significant differences between the data groups are shown as “*” for significance with *p* values < 0.05 or “**” for high significance with *p* values < 0.01. The dashed lines indicate the limits of detection (LODs).

**High neutralizing antibody titers of the S-L1 PVNP-immunized mouse sera.** A plaque reduction neutralization test (PRNT) was used to determine the neutralizing antibody titers of the mouse sera after two or three immunizations with various immunogens (see above) against replications of the vaccinia virus WR strain in cell culture. The results (Figure 4) demonstrated that the sera obtained from mice immunized with the S-L1 PVNPs exhibited high neutralizing antibody titers, reaching 250 after two immunizations or 2400 after three immunizations. These neutralizing antibody titers were 45 folds higher than that of the mouse sera after three immunizations with the S-J1 PVNPs, although the two groups of mouse sera exhibited similar high IgG and IgA titers (Figure 3). The neutralizing antibody titers induced by the S-L1 PVNPs were significantly greater than those elicited by the free L1 antigen, confirming the enhanced immunogenicity of the L1 antigen induced by the polyvalent PVNPs. On the other hand, the low neutralizing antibody titers induced by the S-J1 PVNPs and the free J1 antigen indicate that the J1 protein is not an effective neutralizing antigen. As expected, the mouse sera after immunization with the S_60_ nanoparticles did not show detectable neutralization (Figure 4).

**Figure 5:**
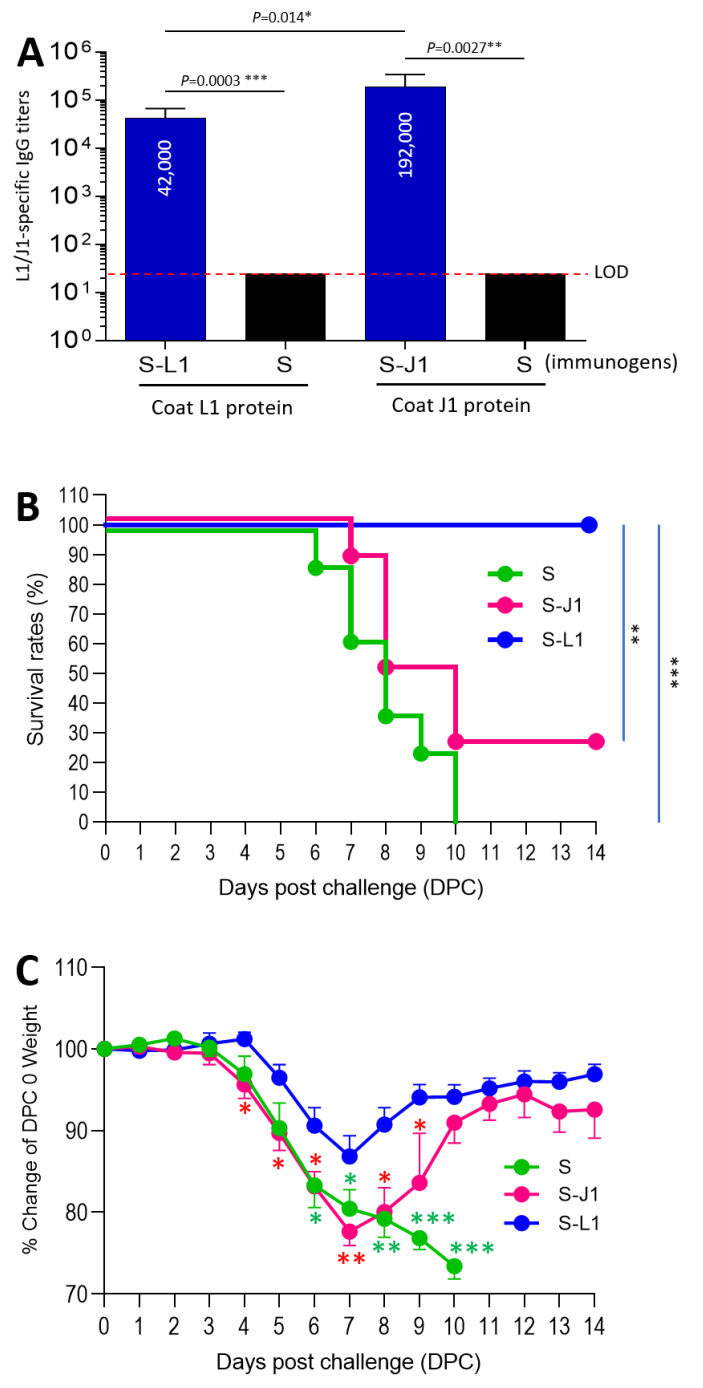
The S-L1 PVNPs protect mice against mortality and body weight loss caused by vaccinia virus challenge. (**A**) L1- (left two columns) and J1-specific (right two columns) IgG responses after three immunizations with the S-L1 PVNP (S-L1) and S-J1 PVNP (S-J1), respectively, using the S_60_ nanoparticles (S) as a negative control. The Y-axis indicates L1/J1-specific IgG titers, while the X-axis shows different immunogens. The dashed lines indicate the limits of detection (LODs). (**B**) Survival curves of three groups of mice after immunization with S-L1 PVNPs (S-L1, blue line), S-J1 PVNPs (S-J1, red line), or S_60_ nanoparticles (S, green line), followed by vaccinia virus challenge. The Y-axis indicates survival rates as a percentage, while the X-axis indicates days post challenge (DPCs). (**C**) Body weight change curves of the same three groups of mice after immunization with S-L1 PVNPs (S-L1, blue line), S-J1 PVNPs (S-J1, red line), or S_60_ nanoparticles (S, green line), followed by vaccinia virus challenge. The Y-axis indicates the body weight change as a percentage, while the X-axis indicates the days post challenge (DPCs). Significant differences between the data sets are shown as “*” for significance with *p* values < 0.05, “**” for high significance with *p* values < 0.01, or “***” for extreme significance with *p* values < 0.001. In (**C**), the green stars indicate significant differences between the S-L1 PVNP (S-L1)-immunized group and the S_60_ nanoparticle (S)-immunized group, while the red stars indicate significant differences between the S-L1 PVNP (S-L1)-immunized group and the S-J1 PVNP (S-J1)-immunized group.

**Protection of the S-L1 PVNPs against body weight loss and mortality caused by vaccinia virus challenge.** Next, we determined the protective efficacy of the PVNP vaccines. After three immunizations, both the S-L1 PVNPs and the S-J1 PVNPs induced high L1/J1-specific serum IgG titers (Figure 5A), among which the S-J1 PVNPs elicited an even greater J1 IgG titer than the L1 IgG titer induced by the S-L1 PVNPs. After the vaccinia virus (WR strain) challenge, the mice vaccinated with the S_60_ nanoparticles began to die on day 6 post challenge (DPC 6), and all the mice died after 10 DPC (Figure 5B, green line). In contrast, all the mice immunized with the S-L1 PVNPs survived the viral challenge, indicating 100% protection against mortality caused by the vaccinia virus challenge (Figure 5B, blue line). However, the protection of the mice vaccinated with the S-J1 PVNPs was low at 25%, as only two out of the eight mice survived the viral challenge (Figure 5B, red line).

Accordingly, mouse body weight change curves indicated that the mice immunized with the S_60_ nanoparticles began to lose weight on DPC 3 and did not recover (Figure 5C, green line). In contrast, the mice immunized with the S-L1 PVNPs started losing weight on DPC 5, reached a maximal weight loss of 13.1% on DPC 7, and then steadily regained weight afterward (Figure 5C, blue line). The mice immunized with the S-J1 PVNPs began losing weight on DPC 3, reaching a maximal weight loss on DPC 7. Subsequently, the survived mice in this group experienced a gradual increase in average body weight (Figure 5C, red line), which was partially attributed to the removal of deceased or euthanized animals that reached the 25% weight loss threshold. Taken together, our study demonstrated that L1 antigen is an excellent vaccine target that is superior to the J1 antigen, and S-L1 PVNPs are a promising vaccine candidate for both vaccinia virus and mpox virus.

## 4. Discussion

In this study, we designed and generated two PVNPs displaying the surface antigens L1 and a membrane-associated protein J1 of vaccinia or mpox virus. We then evaluated the biochemical, biophysical, immune responses, and protective immunity of these PVNPs, with the aim to develop a useful, nanoparticle-based mpox vaccine. The two PVNPs self-assembled from the corresponding fusion protein, S-L1 or S-J1, which were produced at high yields through the use of a mammalian CHO cell expression system. Like other known S_60_ nanoparticle-based PVNPs constructed previously [39,40,41,42], the S-L1 and S-J1 PVNPs are expected to be composed of an inner shell formed by the S domains, resembling the interior layer of the norovirus capsid, and multiple L1 or J1 antigens displayed on the surface. As anticipated, the polyvalent PVNPs substantially increased the immune responses toward the displayed L1 or J1 antigen, eliciting significantly greater L1/J1-specific antibody titers than those induced by the free L1/J1 antigens. Further investigation of mouse sera after two or three immunizations with the S-L1 PVNPs revealed significantly greater serum neutralizing antibody titers against replication of the vaccinia virus in cell culture compared with those of mouse sera after immunization with the S-J1 PVNPs. Consequently, the S-L1 PVNPs provided mice with 100% protection against mortality caused by the vaccinia virus challenge. These data support the conclusion that the readily available S-L1 PVNPs are a promising mpox vaccine candidate.

The significantly higher immune response toward the L1/J1 antigens on the S-L1/S-J1 PVNPs, compared to that induced by the free L1/J1 antigens, should be attributed to the polyvalent presentation of the L1/J1 antigens by the PVNPs, as demonstrated by our previous studies [40,41,42]. It was noted that both L1 and J1 were immunogenic, either as free antigens or on PVNPs. However, the high neutralizing antibody titers (up to 2400) induced by the S-L1 PVNPs and the high protective efficacy (100%) conferred by the S-L1 PVNPs, compared to the low neutralizing antibody titers (up to 53.5) and low protective efficacy (25%) conferred by the S-J1 PVNPs, indicate that the L1 protein is an excellent vaccine target for mpox vaccine development, while the J1 protein is not. This is understandable because L1 is known as an important surface protein in the life cycle of an orthopoxvirus that plays critical roles in the early steps of viral infection, including viral entry through viral–host cell membrane fusion [43]. By binding to the L1 protein, L1-specific antibodies can block the functions of the L1 protein, thus inhibiting viral infection. Consequently, the L1 antigen has been incorporated into several vaccine candidates against orthopoxviruses in the form of DNA vaccines or recombinant protein-based vaccines [32,33,34,35]. A further study mapped the binding region of a neutralizing antibody to a conformational epitope, with Asp35 identified as the key residue [38] (Figure 2J). The study also showed that this epitope is a common site of vulnerability for potent neutralization by a divergent group of antibodies. On the other hand, J1 is known for its roles in virion morphogenesis and plaque formation [44], and these roles may not be required for viral infection.

In addition to the L1 antigen, three additional virion surface proteins of vaccinia virus, namely A27 of IMV, as well as A33 and B5 of EEV, have also been demonstrated to be good vaccine targets against orthopoxviruses [32,33,34,35]. This is evidenced by several multicomponent DNA and recombinant protein vaccine candidates, revealing high neutralization and protection [32,33,34,35]. To further enhance the protective efficacy of our nanoparticle mpox vaccine candidate, we plan to construct three new PVNPs, each of which display the A27, A33, or B5 protein, and evaluate their neutralization and protective efficacy individually using the methods established in this study. The new PVNPs with satisfactory protective efficacy will be selected for combination with the readily available S-L1 PVNPs as a cocktail or multivalent vaccine candidate. We anticipate that such a combined PVNP vaccine targeting three to four important viral neutralizing and protective antigens will provide improved protective efficacy compared to that conferred by the individual PVNP vaccines.

Molecular weight and valence are two important factors affecting the immunogenicity of an antigen. Thus, the relatively low IgG and IgA responses induced by the free L1 and J1 proteins, compared with their S-L1/S-J1 PVNP counterparts, are most likely attributed to their single valence and small molecular weights (<20 kDa), in contrast to the polyvalence of the PVNPs with a much greater molecular weight (>2 MDa), highlighting the advantage of the S-L1 PVNP as a vaccine candidate. We observed that the neutralizing antibody titers induced by the free L1 antigen were very low. This difference may also be attributed to possible misfolding and/or the lack of posttranslational modifications, such as myristoylation, in the *E. coli*-produced recombinant L1 protein, as myristoylation is known to occur in the authentic L1 protein [51]. CHO cell-expressed proteins can undergo glycosylation, which might explain the smeared protein bands observed on the SDS–PAGE and Western blot. However, because poxvirus proteins do not undergo the typical secretory trafficking that eukaryotic membrane proteins do, glycosylation should not occur on vaccinia virus-expressed L1 protein. In general, a eukaryotic expression system capable of providing better folding environment and posttranslational modifications to the target protein may be required to produce S-L1 PVNPs. Finally, owing to the observed low neutralizing antibody titers conferred by the free L1 and J1 antigens, we did not include these two proteins in the vaccinia virus challenge experiments with mice to measure the protective efficacy of the S-L1 PVNP vaccine candidate.

Some limitations were noted in this study. First, despite 100% protection against mortality caused by the challenge with vaccinia virus, the S-L1 PVNP-vaccinated mice underwent up to 13% weight loss on DPC 7, indicating that the vaccinated mice are infected by the challenged vaccinia virus and that the S-L1 PVNP vaccine still has space for improvement. To this end, we plan to develop a tri- or quadrivalent PVNP vaccine covering additional A27, A33, and B5 antigens that have also been shown to be good vaccine targets [32,33,34,35], as described above. Second, we did not evaluate in this study the cellular immune response of our vaccines, which should also play an important role against poxviruses [54,55]. This will be investigated in our next study, although our data in this study showed that the PVNP vaccines induced high antibody responses, and that the antibody neutralized replication of vaccinia virus in high titers. The neutralizing antibody should also play a key role in the observed 100% protection against mortality caused by the challenge with vaccinia virus.

## 5. Conclusions

Using the norovirus S nanoparticle as a platform, we have generated two pseudovirus nanoparticles (PVNPs) that display the L1 antigen of the vaccinia virus and the J1 antigen of the mpox virus, respectively, and evaluated them as an mpox vaccine candidate. Our results demonstrated that the S-L1 PVNPs elicited high neutralizing antibody titers in mice against the vaccinia virus and provided 100% protection against mortality caused by vaccinia virus challenge. In contrast, the S-J1 PVNPs induced low neutralizing antibody titers and conferred weak protective immunity in mice. Thus, the readily available S-L1 PVNPs are a promising mpox vaccine candidate worthy of further development.

## Data Availability

The data presented in this study are available in this article.
